# Identification and Characterization of a Novel Diterpene Gene Cluster in *Aspergillus nidulans*

**DOI:** 10.1371/journal.pone.0035450

**Published:** 2012-04-10

**Authors:** Kirsi Bromann, Mervi Toivari, Kaarina Viljanen, Anu Vuoristo, Laura Ruohonen, Tiina Nakari-Setälä

**Affiliations:** 1 Metabolic Engineering, VTT Technical Research Centre of Finland, Espoo, Finland; 2 Technologies for Health Promoting Foods, VTT Technical Research Centre of Finland, Espoo, Finland; 3 Metabolomics, VTT Technical Research Centre of Finland, Espoo, Finland; 4 Business Solutions Management, VTT Technical Research Centre of Finland, Espoo, Finland; Universidade de Sao Paulo, Brazil

## Abstract

Fungal secondary metabolites are a rich source of medically useful compounds due to their pharmaceutical and toxic properties. Sequencing of fungal genomes has revealed numerous secondary metabolite gene clusters, yet products of many of these biosynthetic pathways are unknown since the expression of the clustered genes usually remains silent in normal laboratory conditions. Therefore, to discover new metabolites, it is important to find ways to induce the expression of genes in these otherwise silent biosynthetic clusters. We discovered a novel secondary metabolite in *Aspergillus nidulans* by predicting a biosynthetic gene cluster with genomic mining. A Zn(II)_2_Cys_6_–type transcription factor, PbcR, was identified, and its role as a pathway-specific activator for the predicted gene cluster was demonstrated. Overexpression of *pbcR* upregulated the transcription of seven genes in the identified cluster and led to the production of a diterpene compound, which was characterized with GC/MS as *ent*-pimara-8(14),15-diene. A change in morphology was also observed in the strains overexpressing *pbcR*. The activation of a cryptic gene cluster by overexpression of its putative Zn(II)_2_Cys_6_–type transcription factor led to discovery of a novel secondary metabolite in *Aspergillus nidulans*. Quantitative real-time PCR and DNA array analysis allowed us to predict the borders of the biosynthetic gene cluster. Furthermore, we identified a novel fungal pimaradiene cyclase gene as well as genes encoding 3-hydroxy-3-methyl-glutaryl-coenzyme A (HMG-CoA) reductase and a geranylgeranyl pyrophosphate (GGPP) synthase. None of these genes have been previously implicated in the biosynthesis of terpenes in *Aspergillus nidulans*. These results identify the first *Aspergillus nidulans* diterpene gene cluster and suggest a biosynthetic pathway for *ent*-pimara-8(14),15-diene.

## Introduction

Filamentous fungi produce various bioactive compounds as secondary metabolites [1], [2]. The genes encoding consecutive steps in a biosynthetic pathway of secondary metabolites are often clustered together on the chromosomes [3]. The clustering of such genes, along with multiple genome sequencing projects [4], [5], has facilitated the prediction of new biosynthetic pathways using bioinformatics. Since secondary metabolites are not crucial for the survival of the organism, their production usually remains silent in normal laboratory conditions [1], [6]. As a result, for most cases in which pathways are discovered through bioinformatic analysis, the products remain undetected [1], [4]–[7]. The role of secondary metabolites for the producing organism is often unclear. They are most likely used as chemical signals in communication and defense to enhance the survival of the organism in its ecological niche [8]. For example, activated secondary metabolite production in *Aspergillus nidulans* has been shown to protect the fungus from fungivory [9]. In addition, for many pathogenic fungi, the virulence that has been hypothesized to protect the fungus in an environment with a diverse array of competing organisms [10] is often mediated by secondary metabolites. Nevertheless, in many cases the biological importance of secondary metabolites for fungi is elusive, and hence the conditions triggering the metabolic biosynthesis are unknown [8].

A variety of methods have been used to uncover the products of silent secondary metabolite clusters in filamentous fungi [3], [11]. One approach has been to manipulate the transcriptional control of the genes involved. Transcriptional regulation of fungal biosynthetic genes for many secondary metabolites is carried out by narrow and broad domain transcription factors [12]. AreA, CreA and PacC are well-characterized broad domain regulators in *Aspergillus nidulans*, where they regulate the production of secondary metabolites in response to changes in the environmental nitrogen, carbon and pH, respectively [8]. Another global regulator of secondary metabolite gene clusters, LaeA (Loss of *AflR* Expression), was identified in a screen for *Aspergillus nidulans* mutants unable to produce sterigmatocystin [12]. *AflR,* the positive regulator of aflatoxin and sterigmatocystin biosynthesis, is not expressed in *laeA* mutants. Expression of also other genes in the sterigmatocystin and penicillin gene clusters is downregulated in the *laeA* deletion strain [13]. The overexpression of *laeA* instead activates multiple putative secondary metabolite clusters. Activation of a biosynthetic gene cluster of previously unknown product in *Aspergillus nidulans*, terrequinone A, was identified in a strain overexpressing *laeA* [13], [14]. LaeA has been implicated to function in chromatin remodeling in the subtelomeric regions of fungal chromosomes, where many secondary metabolite clusters are located [15].

In addition to broad domain transcription factors, narrow pathway-specific regulators also take part in the activation of cryptic secondary metabolite gene clusters [16]. Binuclear zinc cluster (Zn(II)_2_Cys_6_) proteins are a group of pathway-specific transcription factors found only in fungi [17]. AflR, the regulator necessary for aflatoxin and sterigmatocystin biosynthetic gene activation, is a characteristic binuclear zinc cluster protein [18]. AflR is encoded within the sterigmatocystin gene cluster, and it binds to 5′-TCG(N5)GCA motifs found in most promoters of sterigmatocystin/aflatoxin biosynthetic genes [12], [18]. Structurally, the Zn(II)_2_Cys_6_–type transcription factors have a well-conserved cysteine rich domain that binds two zinc atoms. This DNA binding domain recognizes CGG triplets in varying orientations within the promoter region of the target genes [17]. Zn(II)_2_Cys_6_–type proteins are typically encoded within the biosynthetic gene cluster for which they positively regulate expression, as is the case for *Aspergillus nidulans* polyketide asperfuranone and PKS-NRPS hybrid metabolites aspyridone A and B [19], [20]. Likewise in *Fusarium verticillioides*, the overexpression of the Zn(II)_2_Cys_6_–type transcription factor residing in the fumonisin gene cluster is able to activate fumonisin production [21].

In *Aspergillus nidulans,* the discovery of unknown products by using transcriptional upregulation of cryptic gene clusters has been shown to be a potential method for finding novel bioactive metabolites [13]. One such class of compounds, the terpenes, is of particular interest because of their many bioactive and pharmaceutical properties [22], [23]. Many pharmaceutical terpenoids have been isolated from plants used in traditional medicine [24], [25], but there is increasing interest toward terpenoids produced by fungi [26], [27].

The objective of the present study was to determine the terpene producing capability of *Aspergillus nidulans*. Earlier reports suggest the existence of at least one terpene gene cluster in *Aspergillus nidulans* [13]. Here we describe the identification and activation of a novel gene cluster that produces the diterpene *ent*-pimara-8(14),15-diene. We show that genomic mining in the prediction of novel secondary metabolite clusters, and the subsequent transcriptional activation of the clusters, serve as a tool for discovering new metabolites and biosynthetic pathways.

## Results

### Genomic Mining Reveals Two Putative Diterpene Clusters in *Aspergillus Nidulans*

Despite earlier published work suggesting that the *Aspergillus nidulans* genome has only one terpene cluster [13], our analysis instead revealed multiple terpene synthase genes potentially located in biosynthetic clusters. The genes with ‘terpenoid synthase’ or ‘terpenoid cyclase’ InterPro [28] domains were searched from the genome of *Aspergillus nidulans* FGSC A4 [4]. We found 26 such genes, and this group of genes was analyzed by using BLASTp [29] homology search to find putative diterpene synthase homologs. Three ORFs, encoded by locus AN1594, AN3252 and AN9314, showed significant homology to known *ent*-kaurene synthases, whereas the AN6810 sequence shared sequence homology with fungal fusicoccadiene synthase [30]. The genomic neighborhood of these four diterpene synthase homologs was screened for zinc binuclear cluster (Zn(II)_2_Cys_6_) proteins, because these are known to positively regulate the genes within the cluster that encodes them [17]–[21]. We also searched for genes encoding putative cytochrome P450 monooxygenases, since these enzymes are many times involved in terpenoid biosynthesis [31]. We found two gene clusters containing all three genes encoding putative terpene synthase, cytochrome P450, and Zn(II)_2_Cys_6_ protein. Both clusters were selected for further analysis. The selected clusters also contained other putative secondary metabolism pathway genes [6], including dehydrogenases, oxidoreductases, and terpene precursor synthase genes.

### Overexpression of *pbcR* Enhances the Transcription of Seven Diterpene Cluster Genes in *Aspergillus Nidulans*

**Figure 1:**
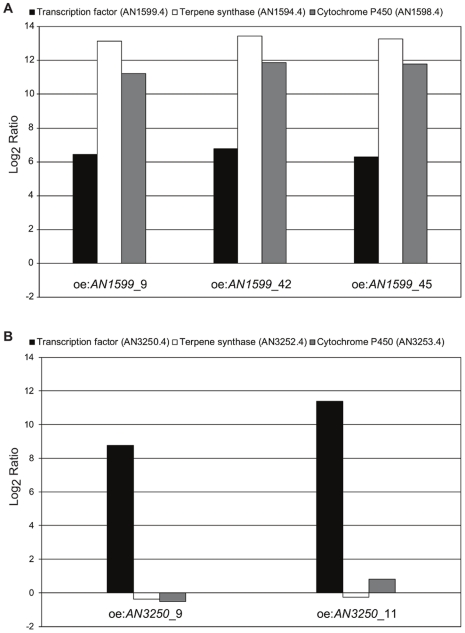
Identification of putative diterpene cluster transcription factor in *Aspergillus nidulans*. Two putative diterpene clusters in *Aspergillus nidulans* were identified by using genomic mining of public databases. Putative transcription factors for the identified clusters were cloned as genomic constructs and overexpressed in *Aspergillus nidulans* FGSC A4. The expression levels of the transcription factors as well as two predicted target genes from each cluster were analyzed by using qPCR. **A)** Overexpression of *pbcR* (AN1599.4) activates transcription of terpene synthase (AN1594.4) and cytochrome P450 (AN1598.4) in three *Aspergillus nidulans* transformant strains (oe:*AN1599*_9, oe:*AN1599*_42 and oe:*AN1599*_45). **B)** Overexpression of the putative transcription factor AN3250.4 fails to alter the transcription of putative terpene synthase (AN3252.4) or cytochrome P450 (AN3253.4) in two *Aspergillus nidulans* transformant strains (oe:*AN3250*_9 and oe:*AN3250*_11).

We discovered a transcriptional regulator, which we named Pimaradiene Biosynthetic Cluster Regulator (PbcR). *PbcR* is encoded by the *Aspergillus nidulans* chromosome VII locus AN1599.4 (GenBank accession number: CBF85190.1), and was cloned as a genomic construct with *Aspergillus nidulans gpdA* promoter and transformed into FGSC A4 wild type strain by random integration. Three independent transformant strains oe:*AN1599*_9, oe:*AN1599*_42 and oe:*AN1599*­_45 were obtained from two different transformations. The presence of the overexpression construct was verified by PCR (Figure S1). FGSC A4 and the transformant strains were grown in YES medium, and the expression levels of *pbcR* (AN1599.4), putative terpene synthase (AN1594.4) and cytochrome P450 (AN1598.4) genes were analyzed by using quantitative real-time PCR (qPCR). As expected, the transcription of *pbcR* was clearly elevated in all three transformant strains compared to FGSC A4; with qPCR analysis demonstrating an 86-fold, 109-fold and 79-fold increase in expression in oe:*AN1599*_9, oe:*AN1599*_42 and oe:*AN1599*­_45, respectively (Figure 1A). The different strain-specific levels of *pbcR* overexpression may have been due to a number of factors including the possibility of overexpression constructs integrating into different portions of the genome or varying *pbcR* copy numbers. For putative terpene synthase gene we observed a 9,000 to 11,000-fold increase in expression in the three transformant strains. For putative cytochrome P450 we observed a 2,400 to 4,500-fold increase in expression (Figure 1A). These results suggest that PbcR is a positive regulator for the diterpene metabolite cluster genes. The strain with the highest transcription of *pbcR*, oe:*AN1599*_42 was selected for further analysis and named oe:*PbcR*.

The putative transcriptional regulator gene at chromosome VI locus AN3250.4 (GenBank accession number: CBF83099.1) was also cloned and overexpressed in FGSC A4. Two isolated transformant strains oe:*AN3250*_9 and oe:*AN3250*_11 were analyzed by using qPCR. Although we detected a 431-fold and 2680-fold increase in expression of the putative transcription factor in oe:*AN3250*_9 and oe:*AN3250*_11, respectively, no significant upregulation of the two target genes for this cluster was observed (Figure 1B). In sum, these results suggest that in contrast to *pbcR*, AN3250.4 overexpression alone does not activate its own putative terpene cluster. However, regulation of AN3250.4 activity at the post-translational level cannot be ruled out.

**Figure 2:**
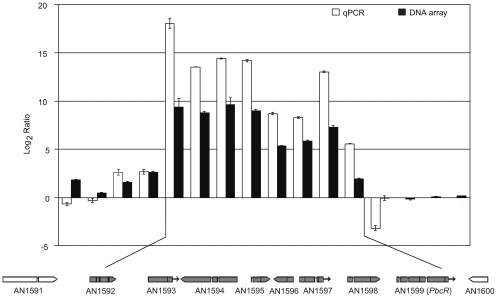
Expression analysis defines the borders of the PbcR activated diterpene cluster in *Aspergillus nidulans*. Transcription factor *pbcR* was overexpressed in *Aspergillus nidulans* strain FGSC A4 (oe:*PbcR*). FGSC A4 and oe:*Pbc*R were grown to their early exponential growth phase in YES-medium. In both strains, the expression levels of 13 genes in the predicted cluster area were measured with qPCR. The fold-change in expression was calculated (white bars). Error bars represent standard error of the mean (SEM, n = 9) for three individual samples with three technical replicates each. The transcriptome of the *pbcR* overexpression strain and the FGSC A4 wild type strain was analyzed by using DNA array and fold differences in expression calculated (black bars). Error bars represent SEM (n = 12) for two cultures with three replicates each; and, each array included duplicate probes. DNA array data represent the comparison of the mean values using confidentiality level 99% with p-values ≤ 0.01 in student’s t-test. Both qPCR as well as DNA array analysis show that overexpression of *pbcR* (AN1599.4) leads to significant upregulation of seven genes in the predicted diterpene cluster area. Predicted genes in the cluster are GGPP-synthase (AN1592.4), HMG-CoA reductase (AN1593.4), diterpene synthase (AN1594.4), elongation factor 1-gamma (AN1595.4), short-chain dehydrogenase (AN1596.4), conserved hypothetical protein (AN1597.4), cytochrome P450 (AN1598.4), and Zn(II)_2_Cys_6_–type transcriptional regulator *pbcR* (AN1599.4). Chromosomal area has been adapted from Aspergillus Genome Database [19] showing 27 kb from *Aspergillus nidulans* FGSC A4 chromosome VII positions 1275000 to 1302000 (upregulated genes highlighted in gray).

To define the borders of the biosynthetic cluster, expression of 13 putative cluster genes was analyzed by using qPCR in oe:*PbcR* and FGSC A4. The expression of these genes in the wild type strain was very low, whereas a massive upregulation of seven adjacent genes was seen in oe:*PbcR* (Figure 2). The highly upregulated genes were homologous to GGPP synthase; AN1592.4 (307,000-fold), HMG-CoA reductase; AN1593.4 (12,000-fold), diterpene synthase; AN1594.4 (21,700-fold), translation elongation factor γ; AN1595.4 (19,000-fold), short-chain dehydrogenase; AN1596.4 (420-fold), hypothetical protein with some similarity to methyltransferase; AN1597.4 (310-fold), cytochrome P450; AN1598.4 (8,400-fold) and Zn(II)_2_Cys_6_ –type transcription factor; AN1599.4 (50-fold). Expression levels of five other putative cluster genes were not as significantly altered by the overexpression of *pbcR* (Figure 2). Taken together, these data suggest that the predicted diterpene cluster consists of eight adjacent genes on *Aspergillus nidulans* chromosome VII in the region AN1592.4 to AN1599.4.

### Overexpression of *pbcR* Leads to Widespread Changes in the Transcriptome of *Aspergillus Nidulans*

DNA array analysis was used to analyze the transcriptome of both oe:*PbcR* and FGSC A4. Since most secondary metabolites are produced after the fungus has completed its initial growth phase [32], samples for the expression analysis were taken at the early exponential growth phase when secondary metabolite production for the wild type fungus was expected to be very low. Both strains were grown in YES medium. The eight terpene cluster genes identified by qPCR also displayed similar enhanced expression when assessed by using DNA array analysis. In fact, when compared with FGSC A4, the most abundant transcripts in oe:*PbcR* were the seven PbcR target genes of the predicted terpene cluster, including: AN1592.4 (673-fold), AN1593.4 (439-fold), AN1594.4 (785-fold), AN1595.4 (513-fold), AN1596.4 (41-fold), AN1597.4 (58-fold) and AN1598.4 (156-fold). The DNA array data also confirmed the overexpression of *pbcR* (4-fold) compared to FGSC A4 (Figure 2, Table S1).

**Figure 3:**
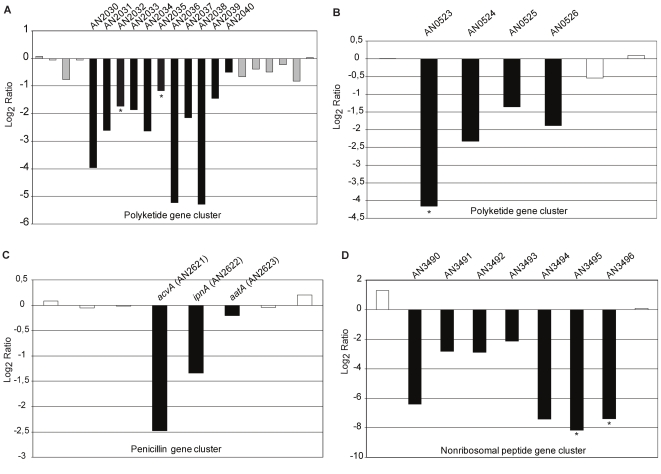
Overexpression of *pbcR* leads to downregulation of four secondary metabolite clusters in *Aspergillus nidulans*. Diterpene cluster transcription factor *pbcR* was overexpressed in *Aspergillus nidulans* strain FGSC A4 (oe:*PbcR*). FGSC A4 and oe:*Pbc*R were grown to their early exponential growth phase in YES-medium. The transcriptome of the oe:*PbcR* and FGSC A4 was analyzed by using DNA array and fold differences in expression calculated. DNA array data represent the comparison of the mean values using confidentiality level 99% with p-values ≤ 0.01 in student’s t-test. Downregulation of four putative gene clusters was seen in oe:*PbcR*. **A)** Shown are expression ratios (oe:*PbcR* to FGSC A4) for genes on chromosome VII in the region AN2030.4 to AN2040.4 (black bars) encoding a putative polyketide gene cluster with polyketide synthase genes AN2032.4 and AN2035.4 (asterisks). **B)** Shown are expression ratios (oe:*PbcR* to FGSC A4) for genes on chromosome VIII in the region AN0523.4 to AN0527.4 (black bars) encoding a putative polyketide gene cluster with polyketide synthase gene AN0523.4 (asterisk). **C)** Shown are expression ratios (oe:*PbcR* to FGSC A4) for genes on chromosome VI in the region AN2621.4 to AN2623.4 (black bars) encoding a penicillin gene cluster with genes *acvA* (AN2621.4), *ipnA* (AN2622.4) and *aatA* (AN2623.4). **D)** Shown are expression ratios (oe:*PbcR* to FGSC A4) for genes on chromosome II in the region AN3490.4 to AN3496.4 (black bars) encoding a putative nonribosomal peptide cluster with two nonribosomal peptide synthases AN3495.4 and AN3496.4 (asterisks).

In addition to the predicted target cluster genes, the expression of a number of secondary metabolite synthase genes was altered in oe:*PbcR*. The polyketide synthase participating in penicillin biosynthesis, *acvA* (AN2621.4), was 5.7-fold downregulated in oe:*PbcR* (Figure 3A, Table S2). Additionally three other putative polyketide synthase genes; AN0523.4 (17.8-fold) (Figure 3B), AN2032.4 (3.3-fold) and AN2035.4 (2.5-fold) (Figure 3C) were downregulated in oe:*PbcR*. Interestingly, many genes adjacent to these synthases were also downregulated, suggesting that the penicillin and two putative polyketide gene clusters are downregulated in the strain overexpressing *pbcR* (Figure 3 A–C). One putative nonribosomal peptide cluster was also dowregulated in oe:*PbcR* (Figure 3D). A significant decrease in expression was seen for the two nonribosomal peptide synthases (NRPS) of this cluster; AN3495.4 (285-fold) and AN3496.4 (167-fold) (Figure 3D). These data demonstrate that activation of the terpene cluster in oe:*PbcR* is associated with changes in the transcriptome of *Aspergillus nidulans* including downregulation of four secondary metabolite clusters. Chromosomal locations of the synthases from this study are shown in Figure S2.

### Activation of the Terpene Cluster Results in *ent*-pimara-8(14),15-diene Biosynthesis in *Aspergillus Nidulans*

**Figure 4:**
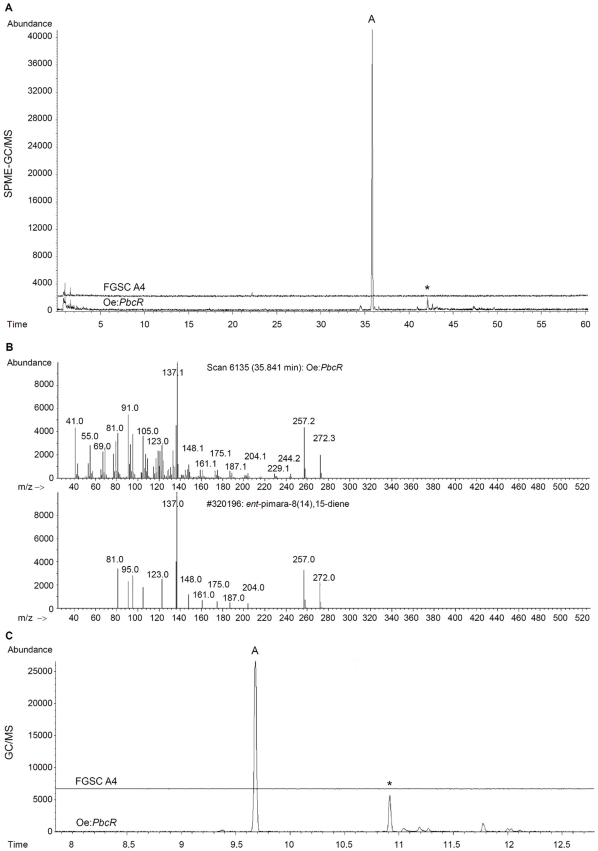
Overexpression of *pbcR* leads to production of *ent*-pimara-8(14),15-diene in *Aspergillus nidulans*. Diterpene cluster transcription factor *pbcR* was overexpressed in *Aspergillus nidulans* FGSC A4 (oe:*PbcR*). The product composition of the oe:*PbcR* and wild type strain (FGSC A4) was analyzed by using gas chromatography mass spectrometry (GC/MS). Fungal cultures were grown to their exponential growth phase in YES medium. **A)** Cultures were analyzed for the production of diterpene compounds using solid phase micro extraction (SPME)-GC/MS analysis as described in Materials and Methods. One prominent peak (labeled A) was observed in the chromatogram of oe:*PbcR*, but no products were detected in FGSC A4. **B)** Product peak A was identified as *ent*-pimara-8(14),15-diene by comparison of its mass spectrum to Palisade Complete 600K Mass spectral library compounds. **C)** Cells from oe:*PbcR* and FGSC A4 were extracted with hexane:ethyl acetate (1∶1) and extracts subjected to GC/MS. As with SPME-GC/MS, one prominent peak (A) was detected. Product peak A was again identified as *ent*-pimara-8(14),15-diene (data not shown).

To identify potential compounds produced in oe:*PbcR*, we analyzed the strains by using solid phase microextraction gas chromatography mass spectrometry (SPME-GC/MS). This analytical method allows identification of volatile and semi-volatile terpenoids [33]. Oe:*PbcR* and FGSC A4 were grown in complete medium for 44 hours and subjected to SPME-GC/MS analysis without further manipulation. An accumulation of an oe:*PbcR*-specific product was observed (Figure 4A). The mass spectrum for the product peak matched the spectral library compound *ent*-pimara-8(14),15-diene (Figure 4B). Also, the calculated retention index for identified *ent*-pimara-8(14),15-diene was 1943, which is in accordance with previous literature values of 1939–1963 [34]–[37]. To determine if non-volatile compounds were produced by oe:*PbcR*, both the cells and the media from fungal cultures were extracted with hexane:ethyl acetate (1∶1) and polar phase extracts subjected to GC/MS-analysis. The data from the cell extracts were consistent with the SPME-GC/MS analysis (Figure 4A, C) and the oe:*PbcR*-specific product was identified as *ent*-pimara-8(14),15-diene according to its mass spectrum (data not shown). No significant product peaks were detected in the extracts from growth medium (data not shown) suggesting that the *ent*-pimara-8,14(15)-diene is not secreted. Taken together this data demonstrates that an activation of a biosynthetic pathway for a diterpene compound occurs in oe:*PbcR*.

### Reduced Conidia and Increased Fruiting Body Formation in Oe:*PbcR*

**Figure 5:**
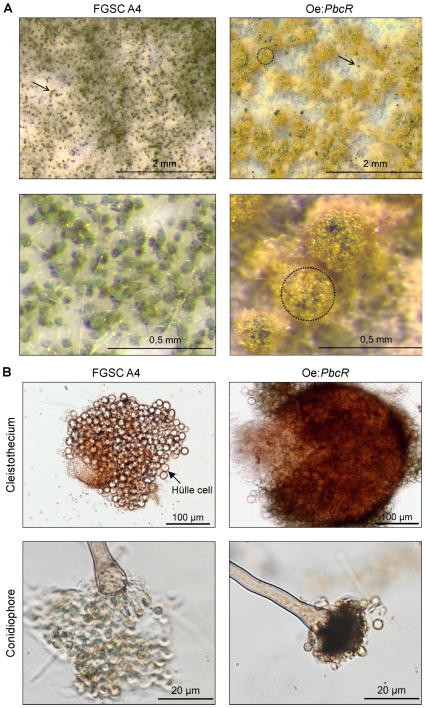
Changes in morphology can be seen in *Aspergillus nidulans* FGSC A4 overexpressing *pbcR* (oe:*PbcR*). Pimaradiene gene cluster regulator *pbcR* was overexpressed in *Aspergillus nidulans* FGSC A4 (oe:*PbcR*). Both wild type FGSC A4 and oe:*PbcR* were grown on potato dextrose plates for 3 days and their morphology studied by microscopy. **A)** Fewer conidiophores (arrows) are seen in oe:*PbcR* compared to FGSC A4. Conidiophore structures in both strains were verified at higher magnification (data not shown). Enhanced sexual fruiting body (cleistothecium, dotted circle) formation can be seen in oe:*PbcR* three-day plate cultures. **B)** The size of cleistothecia in oe:*PbcR* is increased compared to FGSC A4 (upper panels), whereas Hülle cell formation around the fruiting body is similar in both strains. The sterigmata of conidiophores in oe:*PbcR* are darker, and less spores are formed at the tips of the conidiophores than in wild type *Aspergillus nidulans* (lower panels).

**Figure 6:**
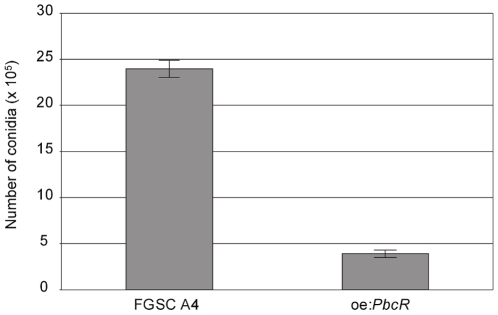
Conidiation is reduced in oe:*PbcR.* FGSC A4 and oe:*PbcR* were grown on potato dextrose plates for three days at 37°C. Spores were quantified from three agar plugs isolated from the PD-plates (average area 85 mm^2^). The number of conidia is 6-fold lower in oe:*PbcR* (average number of conidia 3.9 × 10^5^) compared to FGSC A4 (average number of conidia 23.9 × 10^5^).

Morphological changes were observed in oe:*PbcR* compared with FGSC A4. Specifically, oe:*PbcR* cultures typically grew slower than the wild-type strain (data not shown); and, when grown on plates, oe:*PbcR* appeared yellow whereas FGSC A4 were green (Figure 5A). To characterize the morphological phenotype further, microscopic analysis of the plate cultures was performed, and the conidia were quantified. Sexual fruiting body (cleistothecium) was the predominant structure in oe:*PbcR* and the fruiting bodies were also larger (Figure 5). Hülle cell formation was unaffected in oe:*PbcR*, and the sterigmata of the conidiophores in oe:*PbcR* appeared darker compared to FGSC A4 (Figure 5B). Also, the number of asexual spores (conidia) was reduced 6-fold in oe:*PbcR* compared to FGSC A4 (Figure 6).

### The Sequence Analysis of Terpene Synthase Orthologs Suggests a Bifunctional Role for AN1594.4

**Figure 7:**
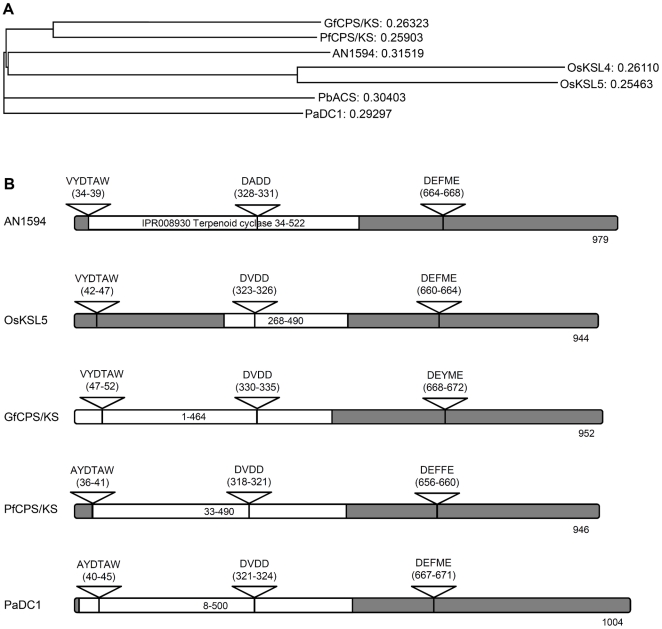
Phylogenetic and primary structure analysis suggests a bifunctional role for *Aspergillus nidulans* pimaradiene synthase (AN1594.4). A) A phylogenetic tree of diterpene synthases was generated by using ClustalW2. Alignment of *Aspergillus nidulans* pimaradiene synthase AN1594 (XP_659198.1) with *Gibberella fujikuroi ent*-kaurene synthase; GfCPS/KS (Q9UVY5.1), *Phaeosphaeria* sp. L487 *ent*-kaurene synthase; PfCPS/KS (O13284.1), *Phoma betae* aphidicolan-16β-ol synthase; PbACS (BAB62102.1), *Phomopsis amygdali* phyllocladan-16α-ol synthase; PaDC1 (BAG_30961.1), *Oryza sativa ent*-pimara-8(14),15-diene synthase; OsKSL5 (NP_001047190.1), and *Oryza sativa syn*-pimara-7,15-diene synthase; OsKSL4 (NP_001052175.1). Phylogenetic tree indicates the similarity of AN1594 to fungal bifunctional diterpene synthases GfCPS/KS, PfCPS/KS, PbACS and PaDC1. AN1594 is also distantly related to known plant pimaradiene synthases OsKSL5 and OsKSL4. The phylogenetic distances are indicated next to the gene names. B) The primary structures of AN1594.4, PbACS, GfCPS/KS, PfCPS/KS and PaDC1 are shown. The inverted triangles indicate conserved motifs in fungal diterpene synthases. The AYDTAW motif is conserved among diterpene cyclases from plants and fungi. The DxDD and DExxE motifs are responsible for the type B cyclization (GGPP to copalyl diphosphate) and type A cyclization (copalyl diphosphate to diterpene), respectively. The InterPro domain IPR008930 “Terpenoid cyclase” is indicated with white bars. The total amino acid length of the proteins is indicated.

The putative terpene synthase gene orthologs were identified using BLASTp search of public sequence databases. AN1594.4 (accession XP_659198.1) showed sequence homology to known bifunctional terpene synthases. Although overall sequence homology was relatively low (Table 1), as is typical for terpene synthase genes generally [38], we identified conserved motifs required for the two cyclization steps carried out by known bifunctional terpene synthases [39] (Figure 7A). A-type cyclization motif, VYDTAW, was identified at position 34–39 and B-type cyclization motif, DEFME, at position 664–668. In addition, the position 328–331 of AN1594.4 encodes a DADD motif, which is conserved among diterpene synthases [38]. A phylogenetic tree was constructed using ClustalW2 multiple alignment analysis of AN1594.4 and the orthologous genes from other fungi, as well as known pimaradiene synthase genes from *Oryza sativa* [40]. The analysis demonstrates that AN1594.4 is related to fungal bifunctional diterpene synthases. Although there are no annotated fungal pimaradiene synthases, AN1594.4 is nonetheless distantly related to known pimaradiene synthases from plants (Figure 7B). The data from the sequence analysis supports the conclusion that AN1594.4 encodes a bifunctional diterpene synthase.

**Table 1:** Protein BLAST alignment of AN1594 shows similarity to known fungal diterpene synthases.

Terpene synthase	Accession	Score	Identities	Positives	Coverage
*Aspergillus nidulans* AN1594	XP_659198.1	2042	100%	100%	100%
*Phomopsis amygdali* phyllocladan-16α-ol synthase, PaDC1	BAG_30961.1	674	39%	55%	94%
*Gibberella fujikuroi ent*-kaurene synthase, GfCPS/KS	Q9UVY5.1	629	37%	57%	94%
*Phoma betae* aphidicolan-16β-ol synthase, PbACS	BAB62102.1	598	36%	53%	97%
*Phaeosphaeria* sp. L487 *ent*-kaurene synthase, PfCPS/KS	O13284.1	547	36%	53%	94%
*Oryza sativa* ent-pimara-8(14),15-diene synthase, OsKSL5	NP_001047190.1	63.9	28%	45%	25%
*Oryza sativa* syn-pimara-7,15-diene synthase, OsKSL4	NP_001052175.1	60.8	28%	45%	23%

## Discussion

Here we show that the Zn(II)_2_Cys_6_-type transcriptional regulator PbcR (Pimaradiene Biosynthetic Cluster Regulator) activates a normally silent secondary metabolite gene cluster in *Aspergillus nidulans*. Upregulation of eight genes in the biosynthetic gene cluster results in *ent*-pimara-8(14),15-diene production in a strain overexpressing *pbcR* (oe:*PbcR*). To our knowledge, *ent*-pimara-8(14),15-diene has not been reported as a natural product in *Aspergillus nidulans*. Previously unknown genes coding for fungal *ent*-pimara-8(14),15-diene synthase, HMG-CoA reductase, and GGPP-synthase are also present in the cluster we describe. We observed morphological changes in the *pbcR* overexpression strains: the number of asexual spores (conidia) is reduced, and the formation and size of sexual fruiting bodies (cleistothecia) is elevated.

The approach we used has been used previously to identify products of other silent metabolite clusters in *Aspergillus nidulans*. For example, the biosynthesis of polyketide asperfuranone and PKS-NRPS hybrid metabolites aspyridone A and B was activated with the overexpression of their pathway-specific transcription factors [19], [20]. The pimaradiene gene cluster upregulated in oe:*PbcR* was previously shown to be one of the putative secondary metabolite clusters upregulated in a *laeA*-overexpressing *Aspergillus nidulans* (OE::*laeA*) [13]. Bok *et al.* [13] demonstrated the upregulation of putative short-chain dehydrogenase (AN1596), cytochrome P450 (AN1598), GGPP-synthase (AN1592), HMG-CoA reductase (AN1593) and terpene synthase (AN1594) in OE::*laeA*. The expression levels of the genes in the pimaradiene gene cluster are different in OE::*laeA* compared to what we observe for oe:*PbcR.* For example, in OE::*laeA*, the expression levels of GGPP-synthase, HMG-CoA reductase and terpene synthase genes were fairly low; and, three cluster genes showed no increase in expression [13]. In contrast, highly elevated expression of eight cluster genes was seen in oe:*PbcR*, and the expression of *laeA* itself was not changed. This suggests that the activation of the diterpene cluster we identified can be differentially regulated by both PbcR and also LaeA. Since multiple secondary metabolite clusters are activated in the *laeA*-overexpressing strain [13], the upregulation of the putative short-chain dehydrogenase and cytochrome P450 genes of the pimaradiene cluster might be needed for modification of secondary metabolites, rather than *ent*-pimara-8(14),15-diene production *per se*. It would be interesting to investigate if *ent*-pimara-8(14),15-diene is produced in the *laeA*-overexpressing strain.

We identified putative genes for HMG-CoA reductase and GGPP-synthase (AN1593.4; GenBank accession number CBF85179.1 and AN1592.4; GenBank accession number CBF85177.1). These genes have not been previously implicated in isoprenoid precursor biosynthesis in *Aspergillus nidulans*. Other studies have identified HMG-CoA reductase (AN3817.2) and GGPP-synthase (AN0654.2, AN2407.2 and AN8143.2) homologs that have been linked to terpenoid biosynthesis in *Aspergillus nidulans* [41]–[43]. However, HMG-CoA reductase and GGPP-synthase identified in this study may specifically provide precursors for the production of *ent*-pimara-8(14),15-diene. The same phenomenon has been suggested for gibberellin biosynthesis in *Gibberella fujikuroi* [44], where a number of precursor synthase genes function separately in different secondary metabolite pathways. The clustering of the HMG-CoA reductase (AN1593.4) and GGPP-synthase (AN1592.4) with the pimaradiene synthase (AN1594.4) may indicate a need for high precursor production required for the biosynthesis of this particular compound.

Although there are some reports of fungal pimaradiene compounds [45], [46], no specific pimaradiene synthases have been identified in fungi. The diterpene synthase identified in our study (AN1594.4; GenBank accession number CBF85181.1) showed similarity to the known fungal *ent*-kaurene synthases GfCPS/KS and PfCPS/KS from *Gibberella fujikuroi* and *Phaeosphaeria* sp., respectively. These terpene synthases catalyze two sequential cyclization steps from GGPP to *ent*-kaurene via *ent*-copalyl diphosphate intermediate [47], [48]. *Phomopsis amygdali* phyllocladan-16α-ol synthase PaDC1 is also a bifunctional terpene synthase having three conserved amino acid domains responsible for the different cyclisation reactions [39]. The diterpene synthase AN1594.4 contains all of these three conserved sequences suggesting the ability to perform two cyclization reactions.

**Figure 8:**
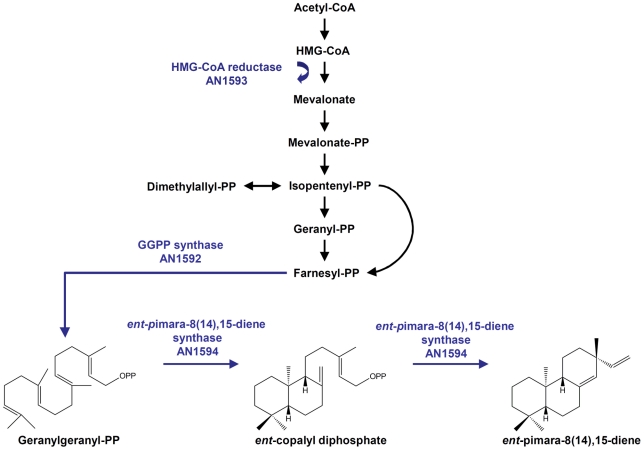
Proposed model of the *ent*-pimara-8(14),15-diene biosynthesis pathway in *Aspergillus nidulans*. PbcR activates key enzymes for pimaradiene biosynthesis. HMG-CoA reductase (AN1593.4) functions as a rate-limiting enzyme in the mevalonate pathway. GGPP-synthase (AN1592.4) provides geranylgeranyl diphosphate precursor for diterpene compounds. Pimaradiene synthase (AN1594.4) is proposed to catalyze two cyclization steps from GGPP to *ent*-pimara-8(14),15-diene via *ent*-copalyl diphosphate intermediate.

Based on the data presented here, we suggest a model for *ent*-pimara-8(14),15-diene biosynthesis in *Aspergillus nidulans*. Specifically, HMG-CoA reductase (AN1593.4) functions in the mevalonate pathway, which produces isoprenoid precursors. GGPP synthase (AN1592.4) is needed in the formation of GGPP, the precursor for diterpenes. Lastly, the two cyclization steps needed to convert GGPP to *ent*-pimara-8(14),15-diene is carried out by pimaradiene synthase (AN1594.4) (Figure 8).

Our analysis revealed four additional genes upregulated in the *Aspergillus nidulans* strain producing pimaradiene. These putative genes encode translation elongation factor 1 gamma (AN1595.4; GenBank accession number CBF85182.1), short-chain dehydrogenase (AN1596.4; GenBank accession number CBF85184.1), hypothetical protein with some similarity to a methyltransferase (AN1597.4; GenBank accession number CBF85186.1), and a cytochrome P450 (AN1598.4; GenBank accession number CBF85188.1). The putative role of these genes in *ent*-pimara-8(14),15-diene biosynthesis is unclear. Cytochrome P450 (AN1598.4), short-chain dehydrogenase (AN1596.4) and methyltransferase (AN1597.4) typically function as decorative enzymes in secondary metabolite biosyntheses [2]. For example, cytochrome P450s add oxygen to the basic terpenoid backbone. This enables chemical modifications of the created hydroxyl group, allowing the formation of a variety of different compounds from the same precursor molecule [31]. Despite the fact that we could not detect the oxidized form of *ent*-pimara-8(14),15-diene in our assay, it is possible that in biological conditions the compound is oxidized to *ent*-pimara-8(14),15-dien-19-oic acid, which is a bioactive diterpene compound predominant in many plant extracts [49], [50].

The expression of two genes (AN1590.4 and AN1591.4) were slightly upregulated in oe:*PbcR.* The expression of these genes was much lower compared to the other putative cluster genes. We identified orthologs of all eight genes included in our cluster in *Neosartorya fischeri*. There, the genes are also adjacent to each other in a putative cluster (Figure S3). But, orthologs of AN1590.4 and AN1591.4 are not present or near this cluster region in *N*. *fischeri*. Thus, we did not include these genes in our putative cluster. However, the possibility that AN1590.4 and AN1591.4 would be under the regulation of PbcR cannot be ruled out.

An increase in fruiting body formation and a reduction in the number of conidia were observed in all *pbcR* transformants. As the integrations were random in nature, it is possible that the phenotype in the transformants is due to insertional mutagenesis. The velvet family of regulators (*veA*, *velB*, *vosA*, and *velC*) participates in sexual fruiting body formation in *Aspergillus nidulans* [51], [52], whereas other genes (e.g., *brlA*, *abaA*, *wetA*, *flbA*, *fluG*, and *fadA*) [53], [54] are implicated in asexual conidiation. All of these genes were similarly expressed in both FGSC A4 and oe:*PbcR*, suggesting they are not regulated by PbcR (data not shown). Siderophore iron metabolism has also been linked to *Aspergillus nidulans* sexual development [55]. Eisendle *et al*. [55] showed that the absence of intracellular siderophore impairs both sexual and asexual reproduction in *Aspergillus nidulans*. The same has been reported for ascomycetes *Cochliobolus heterostrophus* and *Gibberella zeae*, where intracellular siderophores are essential for sexual development [56]. The expression levels of siderophore transporter genes *mirA* and *mirB* [57] were upregulated in oe:*PbcR*. In addition, orthologs of genes implicated in SreA-regulated iron metabolism in *Aspergillus fumigatus* [58] were upregulated in oe:*PbcR* (Table S3). It is tempting to speculate that the sexual phenotype seen in oe:*PbcR* is, at least in part, due to altered regulation of siderophore metabolism genes. Identifying specific genes involved in the altered morphogenesis is beyond the scope of this work given that *Aspergillus nidulans* could conceivably possess as many as 2000 genes that function in some aspect of morphogenesis and development [59].

Overexpression of *pbcR* led to the activation of a pimaradiene gene cluster in *Aspergillus nidulans* FGSC A4. There may be as many as 49 putative secondary metabolite clusters in *Aspergillus nidulans* [13], and we detected downregulation of four of them (penicillin gene cluster, two putative polyketide clusters, and one putative nonribosomal peptide cluster) in oe:*PbcR*. The downregulation of other clusters in the pimaradiene-producing strain might be a way for *Aspergillus nidulans* to ensure sufficient primary metabolites for cell growth, or facilitate the specific production of *ent*-pimara-8(14),15-diene. However, the mechanism for the downregulation of these clusters in oe:*PbcR* is not clear.

We report the first diterpene biosynthetic gene cluster in *Aspergillus nidulans*. Our results affirm the terpene producing ability of *Aspergillus nidulans*, and serve as a proof of principle in finding novel metabolites even in a microbe so widely studied. The results reported here highlight the advantage of using genomic mining in the search for novel biosynthetic pathways.

## Materials and Methods

### Bioinformatic Methods

Putative terpene synthase genes were identified by using InterPro [28] web portal search using domain identifiers IPR008949 ‘Terpenoid synthase’ and IPR008930 ‘Terpenoid cyclase’. To find the potential terpene biosynthetic gene clusters with a positive regulator and characteristic genes for secondary metabolism, InterPro domains IPR001138 ‘Fungal transcriptional regulatory protein’, IPR002403 ‘Cytochrome P450, E-class, group IV’, and IPR001128 ‘Cytochrome P450’ were searched for in 20 kb genomic area around terpene synthase genes.

### *Aspergillus Nidulans* Strains and Growth

*Aspergillus nidulans* strain FGSC A4 (wild type, *veA*+) [60] from Fungal Genetics Stock Center was used in all transformations and experiments as wild type control. Overexpression strains oe:*AN1599_*9, oe:*AN1599_*42 (oe:*PbcR*), oe:*AN1599_*45, oe:*AN3250_*9 and oe:*AN3250_*11 were constructed as described below. Strains were grown in liquid YES-media (2% yeast extract, 4% sucrose) supplemented with 3% gelatin. Transformants were selected on Aspergillus minimal medium (MM) [61] with 200 µg/mL of glufosinate ammonium.

### Construction of Plasmids

Genomic DNA was isolated from FGSC A4 mycelia disrupted with glass beads using standard phenol extraction and ethanol precipitation protocol [62]. DNA was further purified with Qiagen MiniPrep kit. Genomic sequences of AN1599.4 (GenBank: BN001307) and AN3250.4 (GenBank: BN001306) were cloned into the pCR2.1 TOPO (Invitrogen) with SpeI and SpeI and ApaI sites, respectively. Overexpression vector, pKB1, was constructed by adding glufosinate ammonium resistance gene *bar* from pTJK1 [63] into NotI site of modified pAN52-1NotI-vector [64]. *Bar* gene in pKB1 is fused with *Aspergillus nidulans trpC* promoter. AN1599.4 and AN3250.4 genomic sequences were cloned into their respective restriction sites in pKB1 fused with *Aspergillus nidulans gpdA* promoter. All constructs were analyzed by sequencing before transformations. Primers used in PCR are listed in Table S4.

### Transformation

Protoplasting of *Aspergillus nidulans* FGSC A4 was carried out at 30°C in citrate buffer (0.8 M KCl, 0.05 M Na-citrate, pH 5.8) supplemented with 1mM DTT and 1% w/v Hydrolyzing enzymes from *Trichoderma harzianum* (Sigma). Protoplasts were collected by filtration and suspended in 180 µL of cold GTC buffer (1 M glucose, 50 mM CaCl_2_, 10 mM Tris-HCl, pH 5.8). 20 µg of linearized expression plasmid DNA was added and volume adjusted to 200 uL. 50 µL of PEG-solution (25% PEG6000, 50 mM CaCl_2_, 10 mM Tris-HCl, pH 7.5) was added and the suspension incubated on ice for 20 minutes. 2 mL of PEG-solution was added, and the suspension incubated at room temperature for 5 minutes. Protoplasts were plated on selective MM plates in top agar and incubated at 30°C until transformed colonies were visible. Colonies were further grown on selective MM plates and positive colonies verified with PCR.

### Quantitative Real-time PCR Analysis (qPCR)

FGSC A4 and transformant strains were grown in YES medium at 30°C for 42 hours. Due to different germination and/or growth rate of different strains, the conidia were inoculated in varying densities to achieve comparable growth of the cultures at the time of collection. Three individual 100 µL samples from each culture were collected and frozen in liquid nitrogen. Total RNA of the homogenized samples was extracted using Qiagen RNeasy Plant Mini Kit following manufacturer’s suggestions for fungal RNA extraction. Extracted RNA was treated with DNaseI digestion (Qiagen) and quantified using Nanodrop (Thermo Scientific). cDNA synthesis was done with Transcriptor First Strand cDNA Synthesis Kit (Roche). DNA was analyzed by qPCR with LightCycler 480 SYBR Green I Master mix (Roche) on a LightCycler 480 (Roche). All samples were tested in three replicates. Expression levels were normalized to the levels of *β-actin* expression in each sample. Efficiencies for each primer set were calculated, and the expression fold ratios of transformant to FGSC A4 were quantified using pfaffl-equation [65]. Expression levels were checked in similar manner multiple times with consistent results. Primers are listed in Table S4.

### Analysis of Diterpenes by Solid Phase Microextraction Gas Chromatography Mass Spectrometry (SPME-GC/MS) and GC/MS

Conidia of oe:*PbcR* and FGSC A4 were inoculated in varying densities and grown in YES-media at 30°C for 44 hours. SPME-GC/MS was done for cultures with comparable growth. 2 mL of the cultures were transferred into airtight SPME vials. Extraction of volatile and semi-volatile compounds was done at 80°C for 1 hour with preconditioned (250°C, 30 min) 100 µm PDMS fibre (Supelco, USA). Analytes were desorbed during 5 minutes at 250°C in the splitless injector (flow 14.9 mL/min) of the gas chromatograph (Agilent 6890 Series, USA) combined with an MS detector (Agilent 5973 Network MSD, USA) and SPME autosampler (Combipal, Varian Inc., USA). Analytes were separated on BPX5 capillary column of 60 m x 0.25 mm with a phase thickness 1.0 µm (SGE Analytical Science Pty Ltd, Australia). The temperature programme started at 40°C with 1 minute holding, then increased 9°C/min up to 130°C, followed by 2°C/min increase up to 230°C, where the temperature was kept for 1 minute. MSD was operated in electron-impact mode at 70 eV, in the full scan *m/z* 40–550. The ion source temperature was 230°C and the interface was 280°C. Compounds were identified by comparing the mass spectra on Palisade Complete 600 K Mass Spectral Library (Palisade Mass Spectrometry, USA).

For GC/MS analysis, hexane:ethyl acetate (1∶1) extracts were prepared. FGSC A4 and oe:*PbcR* cells were homogenized with mortar and pestle in liquid nitrogen. 2 g of homogenized cells and 100 mL of growth media were ultrasonically extracted for 1 hour with 20 mL of hexane:ethyl acetate (1∶1) 1µL of solvent phase, concentrated by evaporation, was analyzed by using GC/MS (Agilent 6890 Series, USA combined with Agilent, 5973 Network MSD, USA and Combipal injector, Varian Inc., USA). Analytes were injected on split mode (10∶1) and separated on HP-1 capillary column (25 m x 0.2 mm) with a phase thickness 0.33 µm (Agilent, USA). Helium was used as carrier gas, 1.3 mL/min. The temperature program started at 100°C with 0.5 minute holding time, then increased 10°C/min up to 320°C where kept for 25 minutes. MSD was operated in electron-impact mode at 70 eV, in the full scan *m/z* 40–550. The ion source temperature was 230°C and the interface was 280°C. Compounds were indentified with the Palisade Complete 600K Mass spectral library (Palisade Mass Spectrometry, USA). Kovats retention index was determined in relation to a homologous series of n-alkanes (C8–C24) as standards.

### DNA Array Expression Analysis

FGSC A4 and oe:*PbcR* were inoculated in different densities in 50 mL of YES medium. The pH values of each culture were monitored during growth and the mycelia harvested from cultures with pH values ranging from 5.76 to 5.94 indicating the early exponential growth phase of the fungal strains. FGSC A4 was grown for 22 hours and oe:*PbcR* for 26 hours at 37°C. Three RNA extractions were made from two separate culture flasks for both WT and oe:*PbcR*. The quality of RNA was assessed with the standard protocol of Agilent 2100 Bioanalyzer (Agilent Technologies). DNA array chip was designed and manufactured by NimbleGen Systems Inc., Madison, WI USA, using Custom Eukaryotic 12×135K Array format. Sequences for the 10597 transcripts in the DNA array design were downloaded from the Central Aspergillus Data Repository, CADRE [66] via FTP server at Ensembl Genomes browser (ftp://ftp.ensemblgenomes.org/pub/fungi/release4/fasta/aspergillus_nidulans/cdna/Aspergillus_nidulans.CADRE2.4.cdna.all.fa.gz). Expression portion was designed by selecting 6 probes per transcript for 10546 out of 10597 transcripts. Each probe had a replicate for a final expression analysis for total of 126,260 probes. cDNA synthesis of total RNA, probe hybridization, scan and preliminary analysis was performed by NimbleGen Systems Inc., Madison, WI USA, following their standard operating protocol. Normalized DNA array data was further analyzed using the ArrayStar (DNASTAR) software. Expression fold changes were calculated with unpaired, two-tailed, equal variance student’s t-test with 99% significance level, p-value ≤ 0.01. All data are MIAME compliant and the raw data has been deposited in GEO (Accession # GSE32954).

### Conidia Quantification

FGSC A4 and oe:*PbcR* were grown on potato dextrose (PD) plates at 37°C for three days. Three agar plugs were isolated from three PD-plates with a stainless-steel tube with inner diameter of 60 mm (surface area of three plugs is approximately 85 mm^2^). Each plug was homogenized in 500 uL ddH20. The conidial suspension was diluted 1∶10 and spores counted with hemocytometer. The statistical analysis was done with GraphPad InStat using unpaired student’s t-test (p-value <0.0001, n = 9).

### Microscopy

FGSC A4 and oe:*PbcR* were grown on PD plates for three days at 37°C. Stereomicroscope images of untreated samples were taken using Zeiss SteREO DiscoveryV8 microscope equipped with Olympus Soft Imaging Systems DP-25 camera using 8 X magnification. For higher magnification images, conidia were suspended in 20% glycerol and spread to cover slips. Images were taken using Olympus 1X81 microscope equipped with QImaging Retiga-2000R camera. All image visualizations were performed with Olympus Cell P software.

## Supporting Information

Figure S1 **PCR analysis shows the presence of overexpression constructs in** ***pbcR*** **(AN1599.4) transformants.** *Aspergillus nidulans* FGSC A4 was transformed to carry a genomic copy of *pbcR* (AN1599.4) with *Aspergillus nidulans gpdA* promoter. Genomic DNA of FGSC A4 and the overexpression strains (oe:*AN1599*_9, oe:*AN1599*_42 and oe:*AN1599*_45) was purified and the integration of the construct was verified by PCR amplification of a 540 base-pair fragment.
(TIF)

Figure S2 **Chromosomal locations of the secondary metabolite synthases from this study.** The chromosomal location of *Aspergillus nidulans* pimaradiene synthase (AN1594) is shown in red. The chromosomal locations of nonribosomal peptide synthases (AN3495 and AN3496), polyketide synthases (AN2032, AN2035 and AN0523) and isopenicillin A synthetase (*ipnA*, AN2622) downregulated in oe:*PbcR* are shown in blue. Putative diterpene synthase AN3252 is shown in black.
(TIF)

Figure S3 ***Aspergillus nidulans*** **pimaradiene cluster gene orthologs (AN1592.4 to AN1599.4) are found in** ***Neosartorya fischeri*** **.** All eight pimaradiene cluster genes in *Aspergillus nidulans* have orthologous genes clustered in *Neosartorya fischeri*. Figure is adapted from Aspergillus Genome Database [19] using ortholog cluster search.
(TIF)

Table S1 Genes with over 5-fold upregulation in oe:*PbcR* compared to FGSC A4 (p-value ≤ 0.01).
(DOCX)

Table S2 Genes with over 5-fold downregulation in oe:*PbcR* compared to FGSC A4 (p-value ≤ 0.01).
(DOCX)

Table S3 Genes implicated in iron metabolism.
(DOCX)

Table S4 Primers used in this study.
(DOCX)
