# Supplementary figures and images for: Identification and Characterization of a Novel Diterpene Gene Cluster in Aspergillus nidulans
Source: PLoS One. 2012 Apr 10;7(4):e35450. doi: 10.1371/journal.pone.0035450 (PMC3323652; doi:10.1371/journal.pone.0035450)

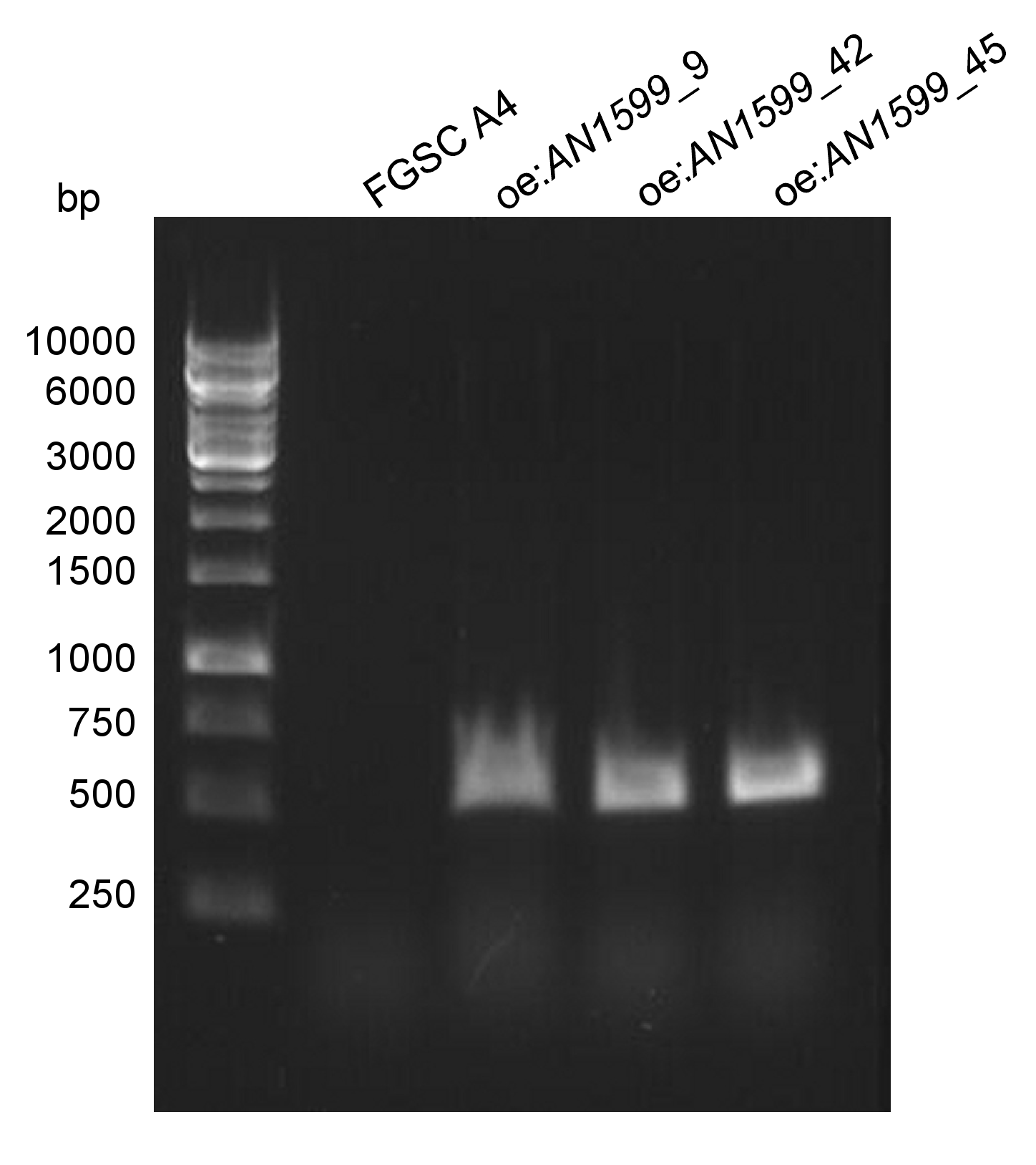

Supplement: Figure S1 — PCR analysis shows the presence of overexpression constructs in pbcR (AN1599.4) transformants. Aspergillus nidulans FGSC A4 was transformed to carry a genomic copy of pbcR (AN1599.4) with Aspergillus nidulans gpdA promoter. Genomic DNA of FGSC A4 and the overexpression strains (oe:AN1599_9, oe:AN1599_42 and oe:AN1599_45) was purified and the integration of the construct was verified by PCR amplification of a 540 base-pair fragment. (TIF) [file pone.0035450.s001.tif]

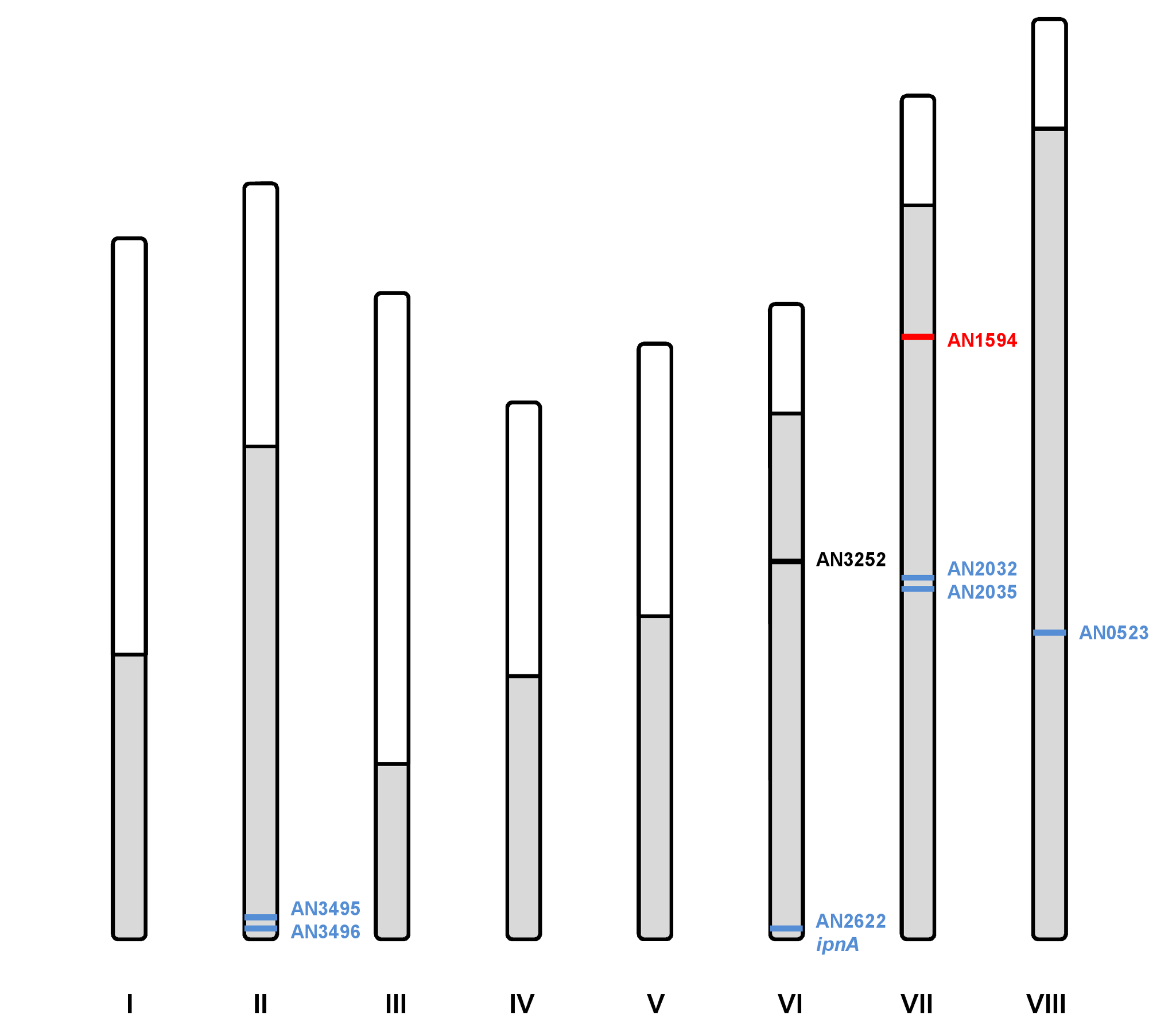

Supplement: Figure S2 — Chromosomal locations of the secondary metabolite synthases from this study. The chromosomal location of Aspergillus nidulans pimaradiene synthase (AN1594) is shown in red. The chromosomal locations of nonribosomal peptide synthases (AN3495 and AN3496), polyketide synthases (AN2032, AN2035 and AN0523) and isopenicillin A synthetase (ipnA, AN2622) downregulated in oe:PbcR are shown in blue. Putative diterpene synthase AN3252 is shown in black. (TIF) [file pone.0035450.s002.tif]

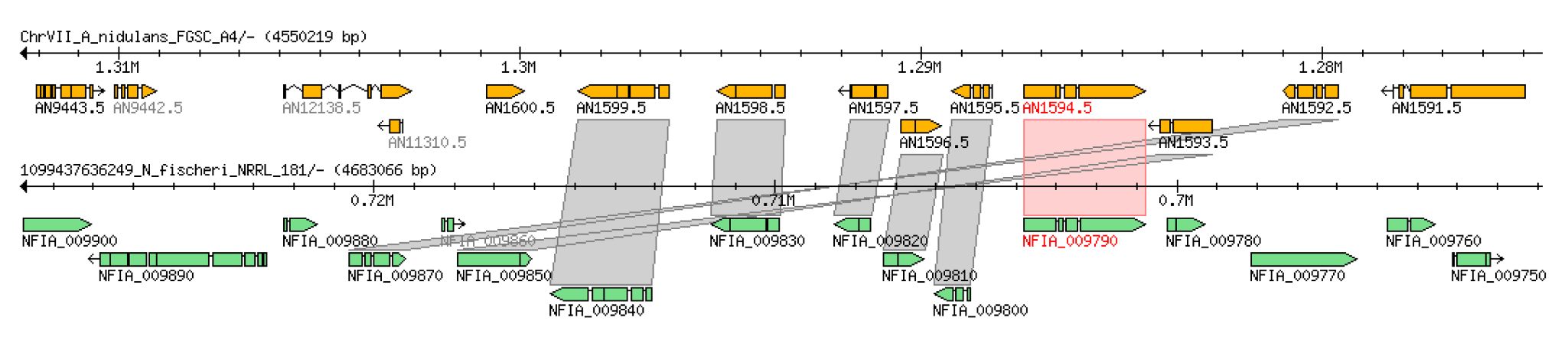

Supplement: Figure S3 — Aspergillus nidulans pimaradiene cluster gene orthologs (AN1592.4 to AN1599.4) are found in Neosartorya fischeri . All eight pimaradiene cluster genes in Aspergillus nidulans have orthologous genes clustered in Neosartorya fischeri. Figure is adapted from Aspergillus Genome Database [19] using ortholog cluster search. (TIF) [file pone.0035450.s003.tif]
