# Supplementary material for: Identification and Characterization of a Novel Diterpene Gene Cluster in Aspergillus nidulans
Source: PLoS One. 2012 Apr 10;7(4):e35450. doi: 10.1371/journal.pone.0035450 (PMC3323652; doi:10.1371/journal.pone.0035450)
Supplement: Table S1 — Genes with over 5-fold upregulation in oe:PbcR compared to FGSC A4 (p-value ≤ 0.01). (DOCX) [file pone.0035450.s004.docx]

Table S1. Genes with over 5-fold upregulation in oe:*PbcR* compared to FGSC A4 (p-value ≤ 0.01)

| Transcript ID^1^ | Fold change (Up) | GENE ID^2^ | Annotation^3^ |
| --- | --- | --- | --- |
| CADANIAT00008227 | 785,30 | AN1594.4 | *ent*-pimara-8(14),15-diene synthase (this study) Ent-kaurene synthase, ent-copalyl diphosphate synthase, Terpene synthase family protein |
| CADANIAT00008225 | 672,99 | AN1592.4 | GGPP-synthase (AFU orthologue) |
| CADANIAT00008228 | 512,68 | AN1595.4 | Elongation factor 1-gamma (translation, protein turnover) |
| CADANIAT00008226 | 438,81 | AN1593.4 | HMG-CoA reductase |
| CADANIAT00008231 | 156,03 | AN1598.4 | Putative cytochrome P450 |
| CADANIAT00008230 | 58,34 | AN1597.4 | Conserved hypothetical protein some similarity to methyltransferase |
| CADANIAT00008229 | 40,57 | AN1596.4 | Short-chain dehydrogenase / NADB Rossmann superfamily domain protein |
| CADANIAT00000113 | 35,05 | AN7322.4 | Putative GPI-anchored protein (AFU orthologue) |
| CADANIAT00010530 | 28,14 | AN11685.4 | FA-hydroxylase and short-chain dehydrogenase domain, similarity to integral membrane protein, bifunctional sterol desaturase/short chain dehydrogenase, short-chain dehydrogenases/reductases family protein, oxidoreductase |
| CADANIAT00002296 | 24,61 | AN0403.4 | Pyridine nucleotide-disulphide oxidoreductase AMID-like, NADP dehydrogenase (AFU) |
| CADANIAT00003030 | 19,87 | AN8539.4 | GNAT-family acyltransferase |
| CADANIAT00002295 | 15,76 | AN0404.4 | AtrH, ABC multidrug transporter |
| CADANIAT00000976 | 15,12 | AN7832.4 | Putative choline dehydrogenase (AFU) |
| CADANIAT00010215 | 14,16 | AN2874.4 | Similarity to alpha-tubulin suppressor (cell division, chromosome partitioning / cytoskeleton, regulator of chromosome condensation) |
| CADANIAT00006016 | 13,38 | AN10558.4 | Putative cAMP-mediated signaling protein Sok1, (AFU orthologue) |
| CADANIAT00001535 | 13,18 | AN1098.4 | Hypothetical protein containing TPR repeat, SEL1 subfamily |
| CADANIAT00000112 | 11,83 | AN7323.4 | Hypothetical protein |
| CADANIAT00000729 | 10,26 | AN7614.4 | Conserved hypothetical protein |
| CADANIAT00010174 | 10,09 | AN2913.4 | Conserved hypothetical protein, some similarity to transglycosylase Slt family and AMP-binding domain protein |
| CADANIAT00009784 | 9,80 | AN3275.4 | Cytochrome P450 |
| CADANIAT00001452 | 9,69 | AN1174.4 | ABC multidrug transporter |
| CADANIAT00007936 | 9,66 | AN8928.4 | ATP-binding cassette multidrug transporter |
| CADANIAT00005862 | 9,62 | AN4575.4 | Hypothetical protein |
| CADANIAT00004818 | 9,55 | AN3883.4 | Putative endo-1,3(4)-beta-glucanase (AFU ortholog) |
| CADANIAT00010385 | 9,52 | AN2729. 4 | MFS transporter |
| CADANIAT00009289 | 9,30 | AN2557.4 | Ado-Met MTase, S-adenosyl mehionine-dependent methyltransferase, SET-domain |
| CADANIAT00000030 | 9,10 | AN7397.4 | Conserved hypothetical protein, COG4995 superfamily domain, Uncharacterized protein conserved in bacteria [Function unknown] |
| CADANIAT00004703 | 8,97 | AN3985.4 | MFS transporter |
| CADANIAT00002962 | 8,75 | AN8477.4 | Xylosidase:Arabinofuranosidase (AFU) |
| CADANIAT00002073 | 8,56 | AN0601.4 | Conserved hypothetical protein, MFS superfamily, membrane transporter, efflux pump antibiotic resistance protein |
| CADANIAT00000940 | 8,51 | AN7800.4 | Siderophone iron transporter mirA (Major facilitator iron regulated transporter A) (Enterbactin permease) |
| CADANIAT00006579 | 8,25 | AN6400.4 | Putative FRE family ferric-chelate reductase (AFU) |
| CADANIAT00002066 | 8,19 | AN0609.4 | Acyl CoA synthetase |
| CADANIAT00000706 | 8,03 | AN10973.4 | Putative citrate synthase (AFU_orthologue) |
| CADANIAT00003239 | 7,77 | AN5239.4 | Conserved hypothetical protein |
| CADANIAT00006138 | 7,59 | AN4325.4 | Hypothetical protein, similarity to putative phosphonopyruvate decarboxylase and BNR/Asp-box repeat domain protein |
| CADANIAT00009436 | 7,55 | AN9165.4 | MFS superfamily protein, MFS multidrug transporter, MFS multidrug resistance protein |
| CADANIAT00007946 | 7,42 | AN8920.4 | Cytochrome b5 reductase, cytochrome b5-like heme / steroid binding domain, FAD-binding oxidoreductase, NAD binding oxidoreductase |
| CADANIAT00009785 | 7,33 | AN10402.4 | Putative FAD-binding oxidoreductase (AFU_orthologue; AFUA_3G03300) |
| CADANIAT00002131 | 7,31 | AN0549.4 | Class V chitinase |
| CADANIAT00001129 | 7,20 | AN9344.4 | ABC multidrug transporter |
| CADANIAT00002130 | 7,06 | AN0550.4 | PGU1-domain protein, endopygalactorunase (cell envelope biogenesis, outer membrane) LysM-domain containing protein, 1.3-beta-glucanase |
| CADANIAT00009048 | 7,02 | AN2349.4 | ATP-binding cassette multidrug transport protein ATRC |
| CADANIAT00010527 | 7,02 | AN8918.4 | Putative Zn(II)2Cys6 transcription factor (Eurofung) |
| CADANIAT00000941 | 7,00 | AN7801.4 | Putative siderophore-degrading esterase |
| CADANIAT00010290 | 6,97 | AN10343.4 | Na+ / phosphate cotransporter, Pho89 |
| CADANIAT00002905 | 6,67 | AN8426.4 | Putative Zn(II)2Cys6 transcription factor (Eurofung) |
| CADANIAT00002960 | 6,60 | AN8475.4 | Eukaryotic phytanoyl-CoA dioxygenase (PhyH) protein. PhyH is a peroxisomal enzyme catalysing the first step of phytanic acid alpha-oxidation. |
| CADANIAT00000227 | 6,43 | AN7220.4 | Conserved hypothetical protein (some similarity to ABC1 transporter / transmembrane protein) |
| CADANIAT00009661 | 6,38 | AN3379.4 | Conserved hypothetical protein |
| CADANIAT00001010 | 6,27 | AN9244.4 | Putative nonribosomal peptide synthase (JCVI) |
| CADANIAT00008224 | 6,26 | AN1591.4 | AAA family ATPase (chaperones, protein turnover, posttranslational modification) |
| CADANIAT00003585 | 6,19 | AN5502.4 | Hypothetical protein, some similarity to carbohydrate kinase, ribokinase family protein, hydrolase of the alpha/beta superfamily family, S9 peptidase, Zn-dependent protease with chaperone function and Forkhead family transcription factor with a major role in the expression of G2/M phase genes (Pichia pastoris) |
| CADANIAT00009859 | 6,15 | AN3207.4 | Neutral amino acid transporter (Eurofung) |
| CADANIAT00003031 | 5,95 | AN8540.4 | Siderophore iron transporter mirB (Major facilitator iron regulated transporter B) (Triacetylfusarinine C permease) |
| CADANIAT00000267 | 5,92 | AN7187.4 | FAD dependent oxidoreductase superfamily (AFU_orthologue) |
| CADANIAT00008385 | 5,91 | AN1738.4 | Conserved hypothetical protein, some similarity to membrane proteins and MFS transporters |
| CADANIAT00006439 | 5,81 | AN8608.4 | Putative D-aminopeptidase (AFU orthologue) |
| CADANIAT00002927 | 5,79 | AN8444.4 | Putative cellulose synthase (Eurofung) |
| CADANIAT00008328 | 5,73 | AN1686.4 | Basic region leucine zipper-domain protein, transcription factor |
| CADANIAT00008463 | 5,41 | AN1814.4 | Conserved hypothetical protein, similarity to glycogen debranching enzyme GlgX, DNA recombination / repair protein, RNA polymerase sigma factor RpoD, ERF family protein and serine/threonine protein kinase |
| CADANIAT00007272 | 5,41 | AN6503.4 | Putative C2H2 transcription factor (Azf1) (AFU_orthologue AFUA_6G05160) |
| CADANIAT00009059 | 5,40 | AN2358.4 | MFS transporter, monocarboxylate permease / transporter |
| CADANIAT00005627 | 5,31 | AN4795.4 | Ras-GEF domain, guanine nucleotide exchange factor for ras-like small GTPases |
| CADANIAT00000833 | 5,23 | AN7702.4 | Putative oligopeptide transporter (AFU_orthologue) |
| CADANIAT00001874 | 5,12 | AN11281.4 | Hypothetical protein |
| CADANIAT00008232 | 3,85 | AN1599.4 | AN1599.4 Zn(II)2Cys6 transcription factor, here named PbcR for Pimaradiene Biosynthetic Cluster Regulator |

^1^ Transcript ID is taken from the Third Party Annotation; TPA; reassembly for *Aspergillus nidulans* FGSC A4.

^2^ Gene ID refers to the locus tag of the annotation version 4.

^3^ Proposed annotations have been generated using closest BLAST and Pfam database hits.
