# Supplementary material for: Identification and Characterization of a Novel Diterpene Gene Cluster in Aspergillus nidulans
Source: PLoS One. 2012 Apr 10;7(4):e35450. doi: 10.1371/journal.pone.0035450 (PMC3323652; doi:10.1371/journal.pone.0035450)
Supplement: Table S2 — Genes with over 5-fold downregulation in oe:PbcR compared to FGSC A4 (p-value ≤ 0.01). (DOCX) [file pone.0035450.s005.docx]

Table S2. Genes with over 5-fold downregulation in oe:*PbcR* compared to FGSC A4 (p-value ≤ 0.01)

| Transcript ID^1^ | Fold change (Down) | GENE ID^2^ | Annotation^3^ |
| --- | --- | --- | --- |
| CADANIAT00005262 | 288,28 | AN3495.4 | Nonribosomal peptide synthase, putative (Eurofung) |
| CADANIAT00005263 | 170,14 | AN3494.4 | Dihydrofolate reductase, esterase lipase superfamily domain, Serine hydrolase FSH1 |
| CADANIAT00005261 | 167,24 | AN3496.4 | Putative nonribosomal peptide synthetase (Eurofung) |
| CADANIAT00005267 | 83,84 | AN3490.4 | Acyl-CoA synthetase |
| CADANIAT00008707 | 39,11 | AN2038.4 | Flavin containing amine oxidoreductase domain protein, similar to FAD dependent oxidoreductase |
| CADANIAT00008704 | 37,37 | AN2036.4 | Putative Zn(II)2Cys6 transcription factor (Eurofung) |
| CADANIAT00009714 | 23,40 | AN3334.4 | NmrA family protein, ergot alkaloid biosynthesis protein, AFUA_2G17970 family |
| CADANIAT00007869 | 19,46 | AN8986.4 | Glycosyltransferase, mannosyltransferase (cell envelope biogenesis outer membrane) |
| CADANIAT00005299 | 18,20 | AN10626.4 | ATP synthase alpha (Broad) |
| CADANIAT00009855 | 18,02 | AN10386.4 | Hypothetical protein |
| CADANIAT00002162 | 17,85 | AN0523.4 | Putative polyketide synthase (JCVI) |
| CADANIAT00006581 | 16,77 | AN6398.4 | UDP-galactose 4-epimerase, putative |
| CADANIAT00008698 | 15,59 | AN2030.4 | Nitroreductase family protein (AFU_orthologue; AFUA_3G03530) |
| CADANIAT00007871 | 14,69 | AN8984.4 | Hypothetical protein, similar to integral membrane proteins |
| CADANIAT00003001 | 14,48 | AN8509.4 | Putative Zn(II)2Cys6 transcription factor (Eurofung) |
| CADANIAT00003781 | 12,95 | AN5315.4 | Conserved hypothetical protein |
| CADANIAT00004741 | 12,81 | AN3952.4 | ABC multidrug transporter (Eurofung) |
| CADANIAT00003695 | 12,43 | AN5397.4 | Conserved hypothetical protein: extracellular laccase (Eurofung) |
| CADANIAT00003706 | 11,41 | AN11477.4 | Hypothetical protein (some similarity to scm-like with MBT domain proteins) |
| CADANIAT00007872 | 11,34 | AN8983.4 | MFS transporter, putative (AFU_orthologue; AFUA_2G02040) |
| CADANIAT00006458 | 11,16 | AN11095.4 | Probable methyltransferase domain, esterase / lipase |
| CADANIAT00006335 | 11,07 | AN8733.4 | Aldo/keto reductase |
| CADANIAT00006410 | 11,06 | AN8637.4 | Catalase A (EC 1.11.1.6) (Spore-specific catalase) |
| CADANIAT00002888 | 10,10 | AN8414.4 | ApdR, Zn(II)2Cys6 transcription factor regulating PKS NRPS hybrid metabolite cluster |
| CADANIAT00005297 | 9,98 | AN10628.4 | SpoC1-C1C protein Fragment |
| CADANIAT00004156 | 9,91 | AN8123.4 | Fructosyl amino acid oxidase, putative |
| CADANIAT00006409 | 8,91 | AN8638.4 | HHE domain protein (AFU_orthologue; AFUA_4G00730) |
| CADANIAT00006502 | 8,77 | AN10812.4 | MFS superfamilly protein, MFS transporter, sugar transporter |
| CADANIAT00006405 | 8,54 | AN8642.4 | NAD binding Rossmann fold oxidoreductase, putative (AFU_orthologue; AFUA_8G04870) |
| CADANIAT00002371 | 8,54 | AN0337.4 | Conserved hypothetical protein with protein kinase-like superfamily domain, methylthioribose kinase domain |
| CADANIAT00006716 | 8,36 | AN6274.4 | short chain dehydrogenase/reductase family (AFU_orthologue; AFUA_7G04540) |
| CADANIAT00001311 | 8,32 | AN1302.4 | NAD dependent epimerase/dehydratase |
| CADANIAT00009753 | 8,13 | AN3305.4 | short chain dehydrogenase/reductase family protein (AFU_orthologue; AFUA_5G14310) |
| CADANIAT00009306 | 7,57 | AN2573.4 | Fatty acid hydroxylase, C4 methyl sterol oxidase |
| CADANIAT00007816 | 7,51 | AN9037.4 | Periplasmic nitrate reductase, putative (AFU_orthologue; AFUA_3G15190) |
| CADANIAT00006337 | 7,51 | AN8732.4 | Putative Zn(II)2Cys6 transcription factor (Eurofung) |
| CADANIAT00005265 | 7,42 | AN3492.4 | Conserved hypothetical protein Similar to zinc-finger proteins |
| CADANIAT00006338 | 7,38 | AN11108.4 | Esterase / lipase (lipid metabolism) |
| CADANIAT00009020 | 7,27 | AN2326.4 | LipA and NB-ARC domain protein (AFU_orthologue; AFUA_3G14650) |
| CADANIAT00005266 | 7,06 | AN3491.4 | MFS superfamily transporter, efflux pump antibiotic resistance protein |
| CADANIAT00007091 | 7,01 | AN5938.4 | Complex I intermediate-associated protein 30 (CIA30), similar to NADH:ubiquinone oxidoreductase complex intermediate-associated protein 30 |
| CADANIAT00006404 | 6,73 | AN8643.4 | bZIP transcription factor (Atf21), putative (AFU_orthologue; AFUA_5G12960) |
| CADANIAT00001291 | 6,63 | AN1322.4 | Dynamin family GTPase, putative (AFU_orthologue; AFUA_4G14300) |
| CADANIAT00004295 | 6,47 | AN8241.4 | Putative chitinase |
| CADANIAT00002992 | 6,47 | AN8504.4 | Nonribosomal peptide synthase GliP-like, putative (AFU_orthologue; AFUA_3G12920) |
| CADANIAT00005296 | 6,36 | AN5086.4 | Conidium-specific protein |
| CADANIAT00005377 | 6,29 | AN5015.4 | Conidiation-specific protein 10 (Eurofung) |
| CADANIAT00007896 | 6,23 | AN8961.4 | Hypothetical protein |
| CADANIAT00008702 | 6,21 | AN2034.4 | Hypothetical protein |
| CADANIAT00002126 | 6,21 | AN0553.4 | Hypothetical protein, some similarity to ABC transporter permease |
| CADANIAT00006501 | 6,19 | AN6468.4 | CorA-like Mg2+ transporter protein |
| CADANIAT00008699 | 6,10 | AN2031.4 | Serine hydrolase (FSH1) Similar to citrinin biosynthesis oxidoreductase |
| CADANIAT00009724 | 5,90 | AN11387.4 | Hypothetical protein |
| CADANIAT00007852 | 5,88 | AN9002.4 | Oxidoreductase,short-chain dehydrogenase/reductase family (AFU_orthologue; AFUA_2G03620) |
| CADANIAT00004009 | 5,84 | AN7989.4 | Phosphatidylserine decarboxylase, putative (AFU_orthologue; AFUA_1G16930) |
| CADANIAT00000741 | 5,83 | AN7624.4 | Putative alpha-galactosidase |
| CADANIAT00009112 | 5,80 | AN11671.4 | Hypothetical protein |
| CADANIAT00005298 | 5,79 | AN10629.4 | Fatty acid desaturase |
| CADANIAT00009848 | 5,66 | AN3214.4 | Sensor histidine kinase/response regulator, histidine kinase A, Histidine kinase-, DNA gyrase B-, and HSP90-like ATPase, Response regulator receiver domain, DNA-binding trascriptional regulator |
| CADANIAT00010505 | 5,57 | AN2621.4 | Hybrid PKS/NRPS, ACV synthetase (ACVS), penicillin biosynthesis |
| CADANIAT00004010 | 5,56 | AN7990.4 | Conserved hypothetical protein |
| CADANIAT00005113 | 5,53 | AN3627.4 | Conserved hypothetical protein |
| CADANIAT00009642 | 5,42 | AN11394.4 | Hypothetical protein |
| CADANIAT00001877 | 5,38 | AN0786.4 | Hypothetical protein |
| CADANIAT00002940 | 5,37 | AN8455.4 | Hypothetical protein with DUF3405-domain, Functionally uncharacterized |
| CADANIAT00002968 | 5,32 | AN11080.4 | DMATS type aromatic prenyltransferase, putative (JCVI) |
| CADANIAT00007599 | 5,31 | AN6803.4 | Pfs, NACHT and WD domain protein (AFU_orthologue; AFUA_7G07100) |
| CADANIAT00007441 | 5,31 | AN6658.4 | Flavin containing polyamine oxidase, putative (AFU_orthologue; AFUA_6G03510) |
| CADANIAT00007085 | 5,30 | AN5943.4 | Conserved hypothetical protein |
| CADANIAT00001705 | 5,20 | AN0948.4 | ABC ATPase, putative (AFU_orthologue; AFUA_1G16440) |
| CADANIAT00001643 | 5,12 | AN1006.4 | Nitrate reductase [NADPH] |
| CADANIAT00003228 | 5,11 | AN5228.4 | NADH:flavin oxidoreductase/NADH oxidase family protein (AFU_orthologue; AFUA_2G04060) |
| CADANIAT00004157 | 5,09 | AN8124.4 | MFS superfamily domain protein |
| CADANIAT00003415 | 5,08 | AN5653.4 | Short-chain dehydrogenase/reductase family protein, putative (AFU_orthologue; AFUA_3G02580) |
| CADANIAT00002161 | 5,03 | AN0524.4 | NmrA family protein |

^1^ Transcript ID is taken from the Third Party Annotation; TPA; reassembly for *Aspergillus nidulans* FGSC A4.

^2^ Gene ID refers to the locus tag of the annotation version 4.

^3^ Proposed annotations have been generated using closest BLAST and Pfam database hits.
