# Supplementary material for: Identification and Characterization of a Novel Diterpene Gene Cluster in Aspergillus nidulans
Source: PLoS One. 2012 Apr 10;7(4):e35450. doi: 10.1371/journal.pone.0035450 (PMC3323652; doi:10.1371/journal.pone.0035450)
Supplement: Table S3 — Genes implicated in iron metabolism. (DOCX) [file pone.0035450.s006.docx]

Table S3. Genes implicated in iron metabolism

| Transcript ID^1^ | Fold change (up) | GENE ID^2^ | Annotation^3^ |
| --- | --- | --- | --- |
| CADANIAT00002296 | 24,60715 | AN0403.4 | Pyridine nucleotide-disulphide oxidoreductase AMID-like, NADP dehydrogenase (AFU) |
| CADANIAT00003030 | 19,87046 | AN8539.4 | GNAT-family acyltransferase |
| CADANIAT00002295 | 15,76098 | AN0404.4 | AtrH, ABC multidrug transporter |
| CADANIAT00000976 | 15,12376 | AN7832.4 | Putative choline dehydrogenase (AFU) |
| CADANIAT00000940 | 8,513795 | AN7800.4 | Siderophore iron transporter mirA (Major facilitator iron-regulated transporter A) (Enterbactin permease) |
| CADANIAT00002066 | 8,187297 | AN0609.4 | Acyl CoA synthetase |
| CADANIAT00000941 | 6,999732 | AN7801.4 | Putative siderophore-degrading esterase |
| CADANIAT00003031 | 5,947138 | AN8540.4 | Siderophore iron transporter mirB (Major facilitator iron-regulated transporter B) (Triacetylfusarinine C permease) |

^1^ Transcript ID is taken from the Third Party Annotation; TPA; reassembly for *Aspergillus nidulans* FGSC A4.

^2^ Gene ID refers to the locus tag of the annotation version 4.

^3^ Proposed annotations have been generated using closest BLAST and Pfam database hits.
