# Supplementary material for: Identification and Characterization of a Novel Diterpene Gene Cluster in Aspergillus nidulans
Source: PLoS One. 2012 Apr 10;7(4):e35450. doi: 10.1371/journal.pone.0035450 (PMC3323652; doi:10.1371/journal.pone.0035450)
Supplement: Table S4 — Primers used in this study. (DOCX) [file pone.0035450.s007.docx]

Table S4. Primers used in this study

| Full length constructs for overexpression | |
| --- | --- |
| Primer name | Sequence |
| AN1599_R | GCACTAGTTCATGTGTTTCTATCTGCGCAA |
| AN1599_F | GCACTAGTATGTACCCGTGGAGTTCGACA |
| AN3250_F | TTGGGCCCATGGTCCCCAACGGCGAGAGA |
| AN3250_R | GCACTAGTCTACGGCACAGGAACAAAATTAG |
| Checking the integration of the constructs | |
| GPD_3’F | CCATCCTTCCCATCCCTTAT |
| AN1599_418R | CAATTCTGCGGACGACAATA |
| AN3250_573R | GCATCGTGGAGGTTCTTGAT |
| Quantitative real-time PCR | |
| Actin_F | GTAAGGATCTGTACGGCAAC |
| Actin_R | AGATCCACATCTGTTGGAAG |
| AN1599_2F | GAATGGCGTAGGACTGTTG |
| AN1599_2R | CGTATCCATAAACTCGGTAATC |
| AN1594_F | TGGAGGTCTTGACAGAGATG |
| AN1594_R | CTCCATTGTGCAGGTAATTC |
| AN1598_F | GACCACTCTCAACAAGATGC |
| AN1598_R | CTGAAATGCGTTCCTTTG |
| AN3250_5F | AACACGTCTTTTCCGCTATC |
| AN3250_5R | GATCAAAGAGTGAGCTTTCG |
| AN3252_6F | TCGCAGAGTGCAAAAGATAG |
| AN3252_6R | TCTGTGACATCGACAAACAC |
| AN3253_F | AGTACTGGGATTTGGCTACG |
| AN3253_R | ATGCACCAGATAGTTCTTCG |
| AN1588_F | GACCAGAGGGGATGTTCTAC |
| AN1588_R | ACTCTGTCCCTGGAATGAAG |
| AN1589_F | CAAGATTCTGCGTCTTCATC |
| AN1589_R | TAATAGGCGAATCTGGTGTC |
| AN1590_F | GATATTGTAGCCCTGTGTGC |
| AN1590_R | ACATGCTGATCACGTAAAGC |
| AN1591_F | AAATAGACGACTCCCACCTG |
| AN1591_R | CCGTCTACCAAGTAGTCACG |
| AN1592_F | TGGGCTTTACTTTCAGATCC |
| AN1592_R | TGTTCTTGGCCCCTTCTC |
| AN1593_F | GTCACGGCGATTTATCTTG |
| AN1593_R | CAGCATTGCCTTTTGAGG |
| AN1595_F | GACCGCAAGTATCTCGTG |
| AN1595_R | TGGGTAAATGCATAACCATC |
| AN1596_F | GCAGCAGAAGAAGCTGAAC |
| AN1596_R | GGGAGGAGGTTCTGAATAAAG |
| AN1597_F | GCATAGCTGGTGGTATATCG |
| AN1597_R | AGCTAGCATTCTTGCGTTC |
| AN1600_F | CATTCATTCGCTTGTAGAGG |
| AN1600_R | CACCTTTTCTGTTTCCACAC |
